# mmS-TCP: Scalable TCP for Improving Throughput and Fairness in 5G mmWave Networks

**DOI:** 10.3390/s22103609

**Published:** 2022-05-10

**Authors:** Geon-Hwan Kim, You-Ze Cho

**Affiliations:** School of Electronic and Electrical Engineering, Kyungpook National University, Daegu 41566, Korea; kgh76@ee.knu.ac.kr

**Keywords:** active queue management, congestion control algorithm, TCP, 5G mmWave

## Abstract

The millimeter-wave (mmWave) band, which can provide data rates of multi-gigabits per second, could play a major role in achieving the throughput goals of 5G networks. However, the high-bandwidth mmWave signal is susceptible to blockage by various obstacles, which results in very large and frequent degradation in the quality of the received signals. TCP, the most representative transport layer protocol, suffers from significant performance degradation due to the very dynamic channel conditions of the mmWave signal. Therefore, in this paper, we propose a congestion control algorithm that guarantees sufficient throughput in 5G mmWave networks and that does not significantly worsen TCP fairness. The proposed algorithm, which is a modification of Scalable TCP (S-TCP) that is designed for high-speed networks, provides a more stable performance than the existing TCP congestion control algorithm in mmWave networks through simple modifications. In various simulation experiments that considered the actual mobile user environment, the proposed mmWave Scalable TCP (mmS-TCP) algorithm demonstrated throughput up to 2.4 times higher than CUBIC TCP in single flow evaluation, and the inter-protocol fairness index when competing with CUBIC flow significantly improved from 0.819 of S-TCP to 0.9733. Moreover, the mmS-TCP algorithm reduced the number of duplicated ACKs by 1/4 compared with S-TCP, and it improved the average total throughput and intra-protocol fairness simultaneously.

## 1. Introduction

Transport layer protocols affect the performance of end-to-end connections over the Internet. Transmission control protocol (TCP), a transport layer protocol used by most applications on the Internet, controls transmission speed by properly adjusting the congestion window (cwnd) indicating the maximum amount of data the sender can transmit based on the congestion control algorithms (CCA) applied to manage network congestion [1]. In addition, when packet loss occurs, reliable packet delivery is ensured by retransmitting and recovering the lost packets based on the acknowledgement (ACK) message delivered from the receiver. With the rapid development of network hardware and the expansion of wireless Internet, various TCP congestion control studies have been conducted to achieve better throughput and low latency [2,3,4,5].

According to the performance requirements of 5G networks, the 5G networks should support gigabits per second (Gbit/s) at their best, and the user bitrate should provide at least 50 megabits per second (Mbit/s) [6]. High-bandwidth millimeter wave (mmWave) communications could play a key role in achieving the throughput goals of these 5G networks. The mmWave band that corresponds to a frequency of 24 GHz or higher provides a wider available bandwidth than do existing mobile communication networks, so higher end-to-end throughput can be expected. Furthermore, it is expected that the demand for high-capacity, low-latency networks caused by the rapid increase in mobile Internet use will be met and resolved through the mmWave band. However, the high-bandwidth wireless channel has a highly variable characteristic whereby extensive signal attenuation occurs even with small obstacles, and the mmWave signal can be greatly attenuated by the human body as well as ordinary buildings [7]. This highly flexible radio channel presents a variety of challenges to existing transport layer protocols and end-to-end applications [8,9,10,11,12,13].

Although the mmWave band is considered a promising technology for achieving the goals of 5G cellular networks with multi-gigabit per second data rates and ultra-low latency, the end-to-end performance experienced by users is ultimately determined by the interaction with the transport layer protocol. In some recent studies, it was noted that the available bandwidth could not be fully utilized due to the large and frequent fluctuations of signals in the mmWave channel, which reduce the end-to-end throughput [14,15]. In order to fully utilize the available bandwidth of the mmWave link, it is necessary to maintain the TCP cwnd value corresponding to the best channel condition, but frequent degradation of the quality of the wireless channel changes the usable capacity of the link. Even if the capacity of the link decreases due to poor radio channel quality, the TCP sender cannot quickly reduce the cwnd and transmits a large number of packets; as a result, the number of packets exceeding the capacity of the wireless link accumulates in the buffer of the base station and generates a long queue. The long queuing delay caused by this bufferbloat [16] problem also increases the round-trip time (RTT), and thus mobile device users experience low quality service as a result.

In previous studies, we analyzed the problems that could arise when TCP operates in a 5G mmWave cellular network, and we conducted related experiments [17,18]. CUBIC [19], the basic TCP congestion control of a representative operating system, could guarantee sufficient throughput when a first-in-first-out (FIFO) queue management scheme was applied to the buffer of the base station. However, the application of FIFO resulted in the creation of a long queue in the radio link control (RLC) buffer, and the RTT greatly increased; this does not achieve the design goal of 5G. Applying CoDel [20], an active queue management (AQM) algorithm, to the base station buffers lowered the end-to-end latency, but due to packet drops by CoDel, CUBIC stayed in the congestion avoidance phase and was unable to fully utilize the link. We evaluated whether the existing TCP CCAs produced similar results and confirmed that Scalable TCP (S-TCP) provides the highest throughput in a single flow [21].

We judged that the 5G design goals could be achieved if high throughput were provided using S-TCP and if low latency were guaranteed through the CoDel scheme. However, because S-TCP increases the cwnd quite dramatically, TCP fairness problems and excessive packet loss may occur. Therefore, in this study, we propose a congestion control algorithm that guarantees sufficient TCP throughput in mmWave networks while providing stability to the network by redesigning the existing S-TCP. The mmWave Scalable TCP (mmS-TCP), redesigned through a simple modification of the cwnd increment/decrement mechanism of S-TCP, can provide excellent performance in mmWave networks in conjunction with CoDel.

The main contributions of this study are as follows:

- The mmS-TCP algorithm simultaneously improves intra-protocol and inter-protocol fairness without degrading the throughput of a single TCP in mmWave networks;
- mmS-TCP greatly enhances network stability through a simple modification of the cwnd increase/decrease mechanism of S-TCP. Therefore, it is easy to deploy and implement;
- By using the mmS-TCP proposed in this study and CoDel scheme together, TCP can guarantee adequate performance in mmWave networks.

The remainder of the paper is organized as follows. In Section 2, we investigate the characteristics of 5G mmWave networks and studies related to TCP in mmWave networks. We introduce the details of the redesigned mmS-TCP in Section 3. Subsequently, we evaluate the mmS-TCP algorithm in various scenarios by conducting a simulation experiment in Section 4. Finally, in Section 5 and Section 6 we discuss our proposed algorithm and conclude the paper.

## 2. Related Work

### 2.1. Characteristics of 5G MmWave Channel

**Figure 1:**
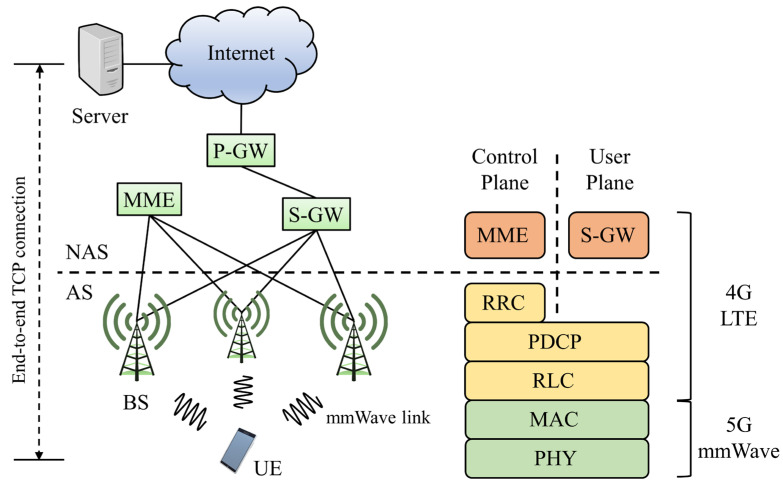
End-to-end TCP connection over 5G mmWave link.

Figure 1 shows a 5G network scenario in which user equipment (UE) communicates with a remote server through a base station (BS in the figure). The BS, which provides mmWave wireless service to mobile devices, communicates with the remote server over an Ethernet connection.

In the wireless network between the UE and BS in Figure 1, packet loss due to the sudden degradation of radio channel quality occurs more often than packet loss due to congestion. TCP can encounter various performance problems in these wireless networks, and many studies have been conducted on the effect of handover and the distance between UEs and base stations on TCP performance in LTE, which is the 4th generation cellular network [22,23].

The mmWave band generally uses a frequency higher than 24 GHz, unlike LTE, which uses the 1.8 GHz band. Thus, the mmWave band, which has a much larger available bandwidth, offers higher throughput than LTE. However, high frequency radio links, where signal attenuation frequently occurs depending on the surrounding environment, present various propagation conditions from the viewpoint of end-to-end connection.

In line-of-sight (LoS) transmission, the maximum bandwidth of a mmWave signal is greatly reduced by obstacles and changed to non-line-of-sight (NLoS) transmission. A mmWave channel with a high maximum bandwidth may have its signal-to-interference-plus-noise ratio (SINR) reduced by 30 dB or more due to signal blocking, which may significantly reduce the available transmission capacity of the link [14]. In addition, the mmWave signal, which is a high frequency signal, consists of a relatively narrow range of cell networks and exhibits more frequent variability than LTE because signal blocking may be caused by small obstacles, even the human body. Therefore, TCP, which must ensure reliable data transfer between end hosts, may experience severe degradation in the mmWave band due to the high maximum bandwidth and frequent channel fluctuations.

### 2.2. Congestion Control in MmWave Network

Network fairness must be considered when designing transport layer protocols. A good TCP CCA provides the best throughput and ensures fairness between TCP flows. However, traditional TCP CCAs do not fully utilize the wide bandwidth of mmWave links and cause excessive latency problems due to the frequent changes in channel state. Therefore, TCP congestion control studies to improve TCP performance in the mmWave environment have been carried out [22,23]. If the TCP sender accurately identifies the state of the mmWave link and adjusts the cwnd, utilization of the link and end-to-end performance improve.

MillProxy [24], a TCP proxy for mmWave cellular networks, divides end-to-end connection into two segments and uses different strategies for wired and wireless communication. It induces throughput improvement by sending data to a larger maximum segment size (MSS) over the mmWave link. MillProxy applies the cross-layer technique to quickly adapt to changes in link state while reducing latency and improving throughput. However, it is very difficult to apply the proxy function to the mmWave base station, and there is the problem that overhead may occur due to the classification operation that divides the end-to-end connection into two segments.

X-TCP [25], a cross-layer CCA that performs congestion control using the parameters of the link layer, finds the optimal cwnd value using the resource information allocated by the base station to the UE and the SINR. X-TCP minimizes latency and quickly adapts to changes in link state without reducing throughput. However, there is the limitation when competes with existing TCP CCAs that the fairness problem may occur, and X-TCP is a cross-layer-based congestion control that is applied only to uplinks.

The design and deployment of new CCAs that are focused solely on improving the performance of mmWave networks can cause fairness issues for existing TCP protocols. For example, [26] showed that RTT fairness issues may arise with changes in channel quality if there is a difference in RTT between TCP flows passing through the mmWave links. Even if multi-path TCP is applied to improve end-to-end throughput and connection robustness, TCP fairness problems may occur [27], so it is necessary to develop a protocol that achieves excellent performance and that maintains fairness in the mmWave environment.

### 2.3. AQM in MmWave Network

The rapid transition from LoS to NLoS due to the presence of various obstacles significantly changes the available capacity of the mmWave band, which greatly affects the performance of the transport layer protocol. If the size of the RLC buffer of the mmWave base station that communicates with the UE is large, the occurrence of buffer overflow is reduced, which thereby reduces the packet loss rate. However, due to the transition from LoS to NLoS, a long queue is quickly created in the buffer, which can lead to a significant delay in end-to-end data delivery [17]. Reducing the buffer size may increase the frequency of buffer overflow and result in frequent packet loss; this may lead to the problem of reduced throughput, as the available bandwidth of the mmWave link cannot be fully utilized.

To take full advantage of the large available bandwidth of the mmWave link, the TCP sender should increase the cwnd to a very large value. In an LoS with good channel conditions, the sending host, which is transmitting a large amount of data through a large cwnd, cannot immediately respond to the temporary deterioration of the channel condition of the mmWave link and continues to transmit excess data. The number of packets exceeding the available capacity accumulates in the buffer of the base station, which creates a long queue and results in the bufferbloat problem [16]. By applying the AQM scheme to mmWave base stations, it is possible to solve the bufferbloat problem and achieve low latency in 5G mmWave networks where frequent and large transitions between LoS and NLoS occur.

Through related studies [14,28] and our previous studies [17,18], we confirmed that combining TCP with a FIFO queue management scheme can guarantee throughput, but RTT is increased excessively in this case, making it impossible to achieve 5G design goals. CoDel can be applied to the RLC buffer to provide low latency, but the problem of throughput degradation occurs because CUBIC TCP does not reach the maximum bandwidth quickly [18].

Therefore, this study aims to provide high throughput by redesigning S-TCP with fast cwnd increase characteristics and to ensure low latency by applying the CoDel queue management scheme. This will help achieve the design goals of 5G.

## 3. Scalable TCP for 5G MmWave Networks

### 3.1. Scalable TCP

S-TCP [29], proposed in 2003 by T. Kelly, provides high throughput and scalability. In the past, NewReno TCP [30] reduced cwnd by half for each packet loss, so link utilization was significantly lower in high bandwidth-delay product (BDP) networks. For example, it takes half an hour, which is a relatively long time, for a NewReno TCP flow with an RTT of 200 ms to reach a bandwidth of 1 Gbps [29]. S-TCP can improve link utilization because it increases cwnd more quickly than does NewReno TCP. Like NewReno TCP, the cwnd increment and decrement mechanism of S-TCP that operates based on packet loss and ACK is as follows:

1. For each ACK received:

(1) cwnd←cwnd+0.01

2. For each packet loss:

(2) cwnd←cwnd−[0.125×cwnd]

After each ACK is received during the RTT, S-TCP increases the cwnd size in proportion to the defined constant (0.01). NewReno TCP increases cwnd by 1/cwnd for each ACK reception, while S-TCP increases cwnd size by 1 MSS if 100 ACKs are successfully received. That is, even in a high-speed network having a large cwnd size, S-TCP increases the delivery rate at a fixed speed. For packet loss events, S-TCP reduces the cwnd size less than the amount reduced by standard TCP congestion control. This reduces the time required for standard TCP to recover to a 10 Gbps link from more than 4 h to less than about 15 s.

### 3.2. Problem Analysis

**Figure 2:**
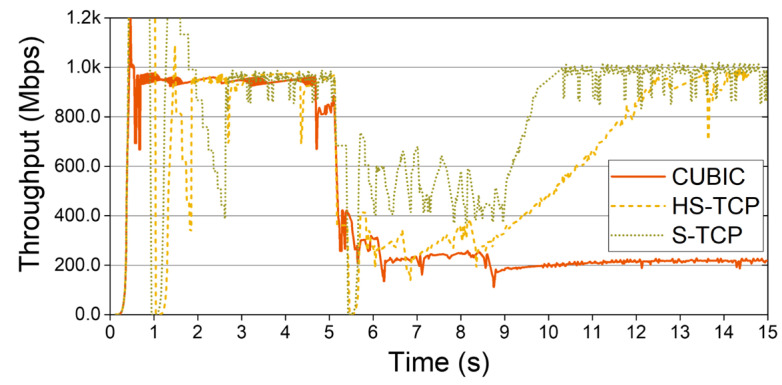
Throughput changes of S-TCP, HS-TCP, and CUBIC TCP.

Before proceeding with this study, we evaluated the performance of the existing TCP congestion control in mmWave networks [17,21]. Based on previous studies, we looked closely at the behavior of S-TCP, as this has not received much attention in other studies. Figure 2 shows the throughput change for a scenario where CUBIC, HighSpeed TCP (HS-TCP) [31], and S-TCP experience one long NLoS. The cwnd increase mechanism of S-TCP ensures a sufficiently fast cwnd increase in transition from NLoS to LoS in the mmWave link. Through the value of the constant in Equation (1), S-TCP can increase the throughput faster than the conventional CCAs can in response to the relaxation of the channel state. On the other hand, TCP CUBIC enters the congestion avoidance phase, slowly increases cwnd for 9 to 15 s, and shows very poor link utilization. The S-TCP reaches its highest bandwidth in about a second, but the HS-TCP does not provide the maximum throughput until after about 13 s.

The results of our preliminary study [21] and Figure 2 show that S-TCP among conventional CCAs can provide sufficient throughput at mmWave network. Because the link utilization of S-TCP in mmWave networks is improved significantly over the default algorithm CUBIC, we judged that S-TCP is worth applying to mmWave networks.

Therefore, we aimed to achieve the design goal of 5G by providing high throughput through S-TCP congestion control and ensuring low end-to-end latency by applying CoDel AQM. However, the increase (more aggressive) and decrease (low reduction) mechanism of S-TCP can undermine TCP fairness and cause throughput imbalance problems between TCP flows. In addition, excessive packet loss may occur due to an aggressive cwnd increase of S-TCP. In Figure 2, S-TCP for 0–3 s performed several slowstart phases due to low cwnd reduction, which could provide serious instability to links in situations competing with other flows.

In this paper, we propose an mmS-TCP congestion control algorithm through a simple redesign of S-TCP that provides sufficient throughput for the 5G mmWave band and can maintain TCP fairness at the same time.

### 3.3. MmWave Scalable TCP

To impose additional stability on S-TCP that can ensure sufficient throughput in mmWave networks, cwnd increase and decrease mechanisms were both modified. The redesign of the increase/decrease mechanisms improves the stability and fairness of the S-TCP in the mmWave network while maintaining the throughput of S-TCP.

#### 3.3.1. Cwnd Increase Mechanism of mm-Scalable TCP

By modifying the cwnd increase mechanism, mmS-TCP can improve TCP fairness. When 100 ACKs are received, the S-TCP increases the size of cwnd by 1 packet. The increase due to the constant value (1 packet) according to 100 ACKs reception provides a faster delivery rate than NewReno. However, a rapid increase in S-TCP may be detrimental to competing TCP flows using other congestion controls. To alleviate the aggression of S-TCP and prevent a decrease in throughput, we redesigned the increasing constant of cwnd as follows:

1. For each ACK received:

(3) $$\mathrm{cwnd}\leftarrow \mathrm{cwnd}+\frac{1}{a}$$

where a increases to 125 by 1 each time ACK is received.

Each time an ACK is received, 1/100 is added to the S-TCP, and when 100 ACKs are received, the cwnd increases by one packet. That is, the increase rate and aggression of S-TCP are determined by the value a of Equation (3). If the vale a is small, the S-TCP increases faster, and if the value a is large, the cwnd increases slowly.

We changed the value of a to gradually increase from 1 to 125 through the counter to reduce the aggression of S-TCP but prevent throughput degradation. The value of a increases by one from 1 to 125 each time an ACK is received. The maximum value of a is set to 125, so that cwnd is increased more slowly than the S-TCP. That is, in the early stages of cwnd increase, the mmS-TCP rises faster than S-TCP, and in the second half of the cwnd increase, it rises more gently than S-TCP to increase fairness with competition flows.

**Figure 3:**
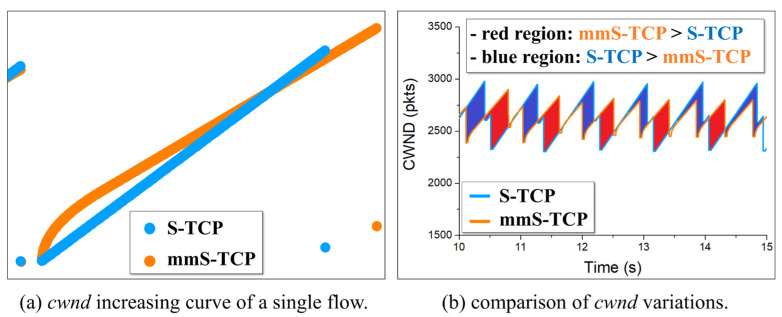
Cwnd change of S-TCP and mmS-TCP.

Figure 3 shows the cwnd increase curves of the S-TCP and the redesigned mmS-TCP. mmS-TCP initially increases the cwnd faster than does S-TCP, and then slowly increases from the time a=125. Compared with the early stage of cwnd increase, the slowly increasing rate allows mmS-TCP to operate more stably.

#### 3.3.2. Cwnd Decrease Mechanism of mm-Scalable TCP

The cwnd decrease mechanism of mmS-TCP aims to reduce excessive packet losses that occur at the beginning of the connection and to give the network stability. For all packet loss situations except retransmission time-out (RTO), S-TCP reduces cwnd by 0.125, as shown in Equation (2). In the slowstart phase, cwnd may increase to a value higher than the actual available capacity of the link in proportion to the bottleneck buffer size, a decrease in cwnd of 0.125 may cause additional RTO and lead to a repetition of the slowstart phase. A repeated slowstart phase can cause many packet losses at the beginning of the TCP connection and adversely affect other flows passing through the same link.

**Figure 4:**
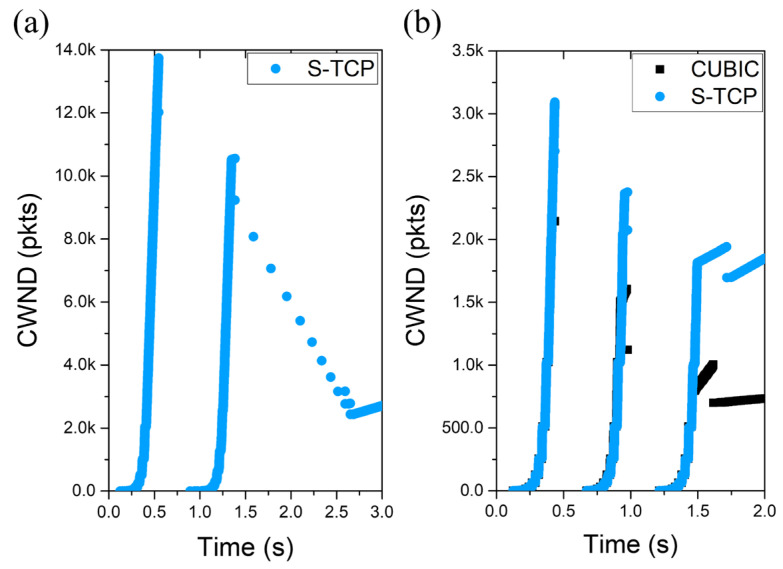
Cwnd change during the slowstart phase: (**a**) single S-TCP; (**b**) CUBIC vs. S-TCP.

Figure 4a shows the slowstart phase of a single TCP flow passing through the mmWave link. S-TCP ends the first slowstart in 0.5 s and reduces cwnd from about 14,000 packets to 12,250 packets. Because the cwnd at this time is also a value that exceeds the bottleneck bandwidth, RTO soon occurs and slowstart is performed again at about 0.8 s. Repeated slowstart phases can cause serious fairness problems for other flows that share links.

Figure 4b illustrates the slowstart phase when both TCP connections begin at the same time in a topology where S-TCP and CUBIC pass the same link. S-TCP has three consecutive slowstart phases due to a small cwnd reduction, and the CUBIC connection is also affected.

**Figure 5:**
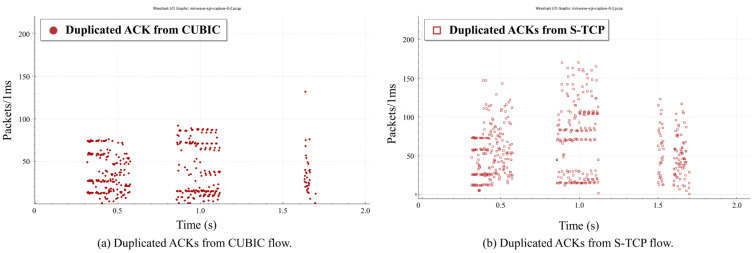
Duplicated ACKs generated during the slowstart phase: (**a**) CUBIC flow; (**b**) S-TCP flow.

Figure 5 is the result of confirming the number of duplicate ACKs generated during the slowstart phase through Wireshark [32]. As illustrated in Figure 5a, several duplicated ACKs occur in the CUBIC flow due to the slowstart phases of the S-TCP flow.

In a situation where two CUBIC flows compete, approximately 11,000 duplicated ACKs occurred in each CUBIC flow during the slowstart phase. This corresponds to 1.4% and 1.1%, respectively, of the total number of packets. However, when CUBIC flows compete with S-TCP, 14,784 duplicated ACKs are generated, representing 2.8% of the total packets. Therefore, in CUBIC, multiple duplicated ACKs are generated by the slowstart repetition induced by S-TCP.

Therefore, in order to eliminate unnecessary slowstart repetition and provide stability to the network, the cwnd decrease mechanism of the S-TCP was changed as follows:

1. For each packet loss:

(4a) cwnd←cwnd−[0.125×cwnd] in the congestion avoidance phase.

(4b) cwnd←cwnd−[0.5×cwnd] in the slowstart phase.

In the congestion avoidance phase, cwnd is reduced by only 12.5% in the same way as the S-TCP. The same cwnd reduction may maintain the link utilization rate during the congestion avoidance, thereby ensuring throughput. However, in order to cope with packet loss occurring in slowstart, the current cwnd size is reduced by half, as in the traditional TCP congestion control (NewReno). This prevents unnecessary slowstart repetitions and ensures fairness with competing flows.

**Figure 6:**
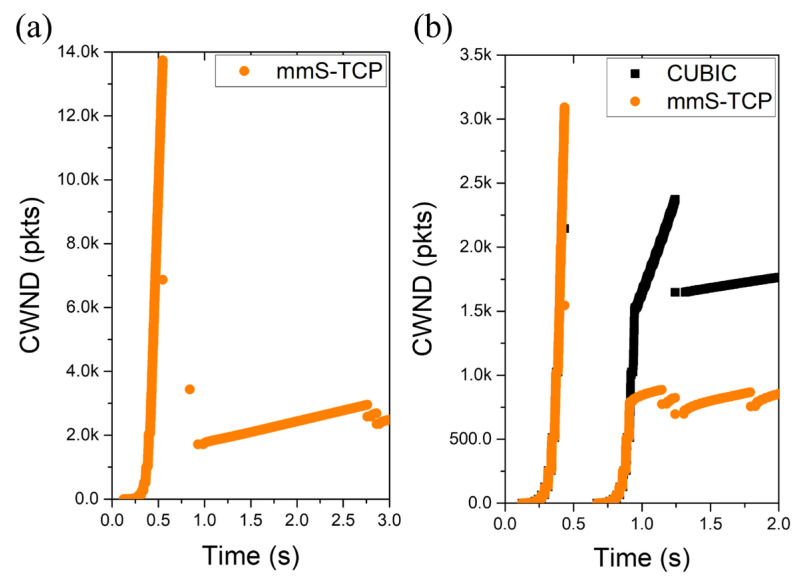
Cwnd change during the slowstart phase: (**a**) single mmS-TCP; (**b**) CUBIC vs. mmS-TCP.

Figure 6 shows the cwnd change during the slowstart phase of the mmS-TCP. In Figure 6a, a single mmS-TCP flow performs congestion avoidance after one slowstart, unlike S-TCP, which performs slowstart twice. Even in a situation that competes with the CUBIC flow, as shown in Figure 6b, the mmS-TCP increases to the slowstart threshold (ssthresh) after one slowstart phase and enters stably into congestion avoidance.

The following results were derived by modifying the cwnd decrease mechanism of S-TCP.

- While repeated slowstart phases by S-TCP caused many duplicated ACKs, mmS-TCP was able to significantly reduce the number of duplicated ACKs at the beginning of the connection;
- Improved network stability and slightly increased throughput;
- After slowstart, mmS-TCP was able to improve fairness with other TCP CCAs because the ssthresh was not too high.

## 4. Evaluations

In this section, we describe the implementation of several 5G mmWave network simulation scenarios to evaluate the performance of the redesigned mmS-TCP. We focus on throughput, number of duplicate ACKs, and TCP fairness.

### 4.1. 5G MmWave Framework

The 5G mmWave framework implemented in ns-3 [33] is built on top of the ns-3 LTE module, LENA [34,35]. LENA is an LTE/EPC network simulator and an open-source framework that enables the creation and testing of various LTE-related applications (design of downlink and uplink schedulers, radio resource management algorithms, inter-cell interference control and UE mobility management, end-to-end service quality tests, etc.). The LENA framework includes elements essential for LTE communication, such as packet data convergence protocol (PDCP), radio resource control (RRC), RLC layer, MAC and PHY layers, and various antenna models. The LTE/EPC protocol structure of the LENA module was utilized in the development of the mmWave simulation module to implement customized PHY and MAC layers for mmWave communication. The implemented 5G mmWave framework [36,37] is currently open for a variety of researchers to design and test new 5G protocols.

**Figure 7:**
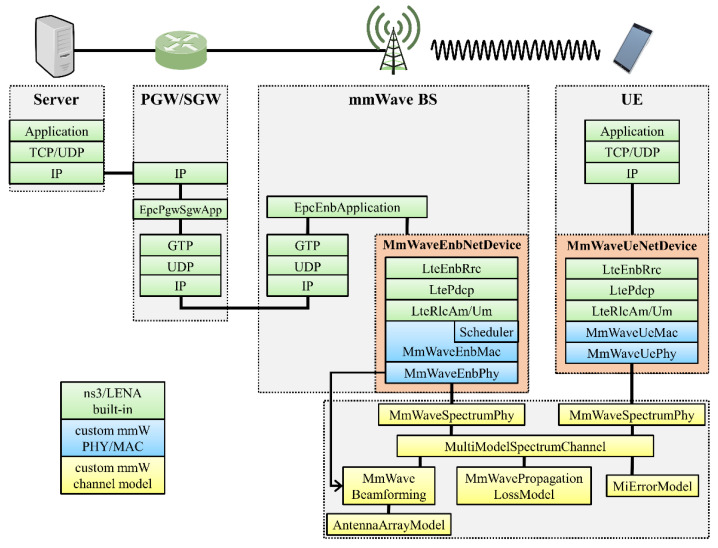
Ns-3-based 5G mmWave module architecture from an end-to-end perspective [36].

Figure 7, introduced by M. Mezzavilla [36], shows the ns-3-based 5G mmWave module architecture from an end-to-end perspective. There are various classes for mmWave, as well as LTE LENA classes. Because the structure and function of each class inherits the existing LTE LENA module, details can be found in the LENA project document and the 5G mmWave framework implementation document [34,35,36,37].

### 4.2. Experimental Scenarios

In this study, various scenarios for TCP connection through 5G mmWave link were constructed to evaluate the performance of the redesigned mmS-TCP. Scenarios for evaluating the performance of single-path TCP over mmWave link are largely divided into three categories. Through these three scenarios, we evaluated the number of duplicated ACKs and the throughput of a single TCP CCA, and the results are covered in Section 4.3 and Section 4.4.

For this, an experiment was performed based on the CCAs implemented in ns-3. S-TCP [29], CUBIC [19], HS-TCP [31], Hamilton TCP (H-TCP) [38], and Yeah [39] algorithms were used as CCAs for performance comparison with mmS-TCP. CUBIC uses a cubic function for cwnd size control. CUBIC is an algorithm that overcomes the problems of fairness and complexity compared to the BIC algorithm and is currently used as a default congestion control in most operating systems. HS-TCP is an algorithm that provides fast cwnd size growth rates with a modified TCP response function when the cwnd size is greater than a specified parameter. H-TCP is an algorithm that increases aggression over time after a previous loss event. Yeah, the only hybrid-based algorithm among CCAs used for evaluation, is a modification of the Scalable TCP, and has two operating modes: high-speed and low-speed.

**Figure 8:**
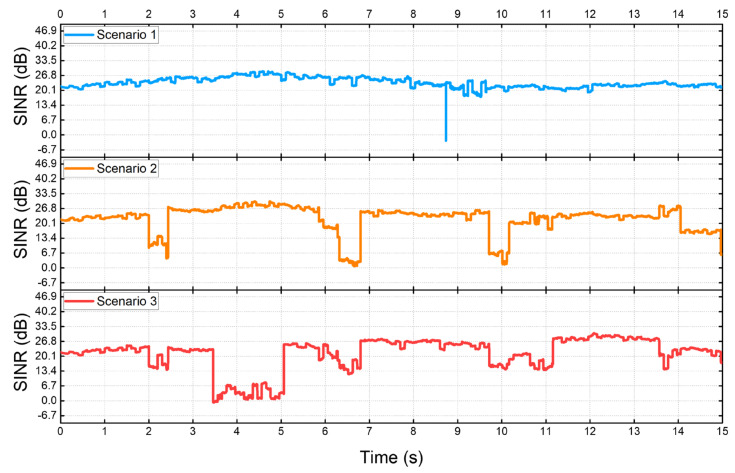
SINR changes in each scenario.

Figure 8 shows a graph of the SINR measured from the UE in each scenario. Scenario 1 assumes a situation in which the SINR decreases by 20 dB due to a single instantaneous outage event. In Scenario 2, it is assumed that the UE experiences a short but deep NLoS of 0.5 s four times. In Scenario 3, it is assumed that a long and large NLoS of about 1.5 s is experienced once, and a short and shallow NLoS four times. Table 1 shows the set values of the network parameters used in each simulation scenario.

### 4.3. Number of Duplicated ACKs

The cwnd decrease mechanism of S-TCP unnecessarily repeated the slowstart phase, resulting in many duplicated ACKs. To examine by how much the number of duplicated ACKs is reduced by the modified cwnd increase/decrease mechanism of mmS-TCP, we performed the single TCP experiments of Scenarios 1–3. The total number of packets and the number of duplicated ACKs in each TCP connection were confirmed using Wireshark.

**Figure 9:**
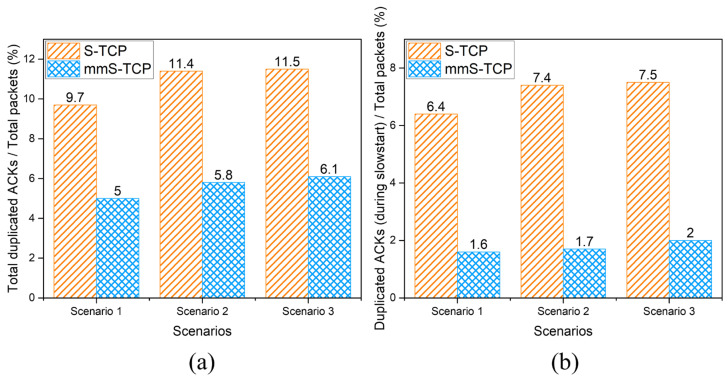
Comparisons of duplicated ACKs generated by S-TCP and mmS-TCP: (**a**) total duplicated ACKs/total packets; (**b**) duplicated ACKs during slowstart phase/total packets.

Figure 9a shows the number of duplicated ACKs generated by S-TCP and mmS-TCP divided by the total number of packets. In each scenario, duplicated ACK packets from S-TCP account for about 10–11% of the total packets. However, mmS-TCP is only about 5–6% of all packets and is reduced by about half compared with S-TCP.

To confirm the performance change due to the modification of the cwnd decrease mechanism, we checked the number of duplicated ACKs that occurred during the slowstart phase of each connection. A single S-TCP enters the congestion avoidance phase after performing a slowstart twice. The time when the slowstart phases end is about 3 s. Therefore, the number of duplicated ACKs generated in the S-TCP connection for 0 to 3 s was summed. In the case of mmS-TCP, because slowstart is performed only once, the number of duplicated ACKs that occurred between 0 s and 1 s was counted.

Figure 9b shows that the proportion of duplicated ACKs occurring in the S-TCP flow during the slowstart phase of 3 s in the entire 15-s simulation experiment is approximately 7%. That is, about 7% of all packets of S-TCP are duplicated ACK packets transmitted during the slowstart phase. In mmS-TCP, about 2% of all packets were found to be duplicated ACKs generated during a relatively short slowstart phase of 0 s to 1 s. As a result of comparing the number of duplicated ACKs for the same 3 s as S-TCP, mmS-TCP showed a significantly lower rate of approximately 1.8%, 2.3%, and 2.6% in each scenario.

Therefore, with the redesign of the cwnd increase/decrease mechanism, mmS-TCP halved the total number of duplicated ACKs compared with S-TCP, and it reduced the number of duplicated ACKs by approximately 1/4 during the slowstart phase.

Table 2 compares the rate of duplicated ACKs to total packets generated by one TCP flow for each CCAs. CUBIC has a significantly lower rate of duplicated ACKs compared to S-TCP and mmS-TCP. However, in Scenario 2 and 3, except for Scenario 1, CUBIC has approximately 40% (for Scenario 2) and 60% (for Scenario 3) lower throughput than mmS-TCP because CUBIC does not fully utilize the available bandwidth. Therefore, mmS-TCP, which aims to provide higher throughput than CUBIC in mmWave networks, shows advantages in terms of throughput improvement.

### 4.4. End-To-End Throughput of Single Flow

**Figure 10:**
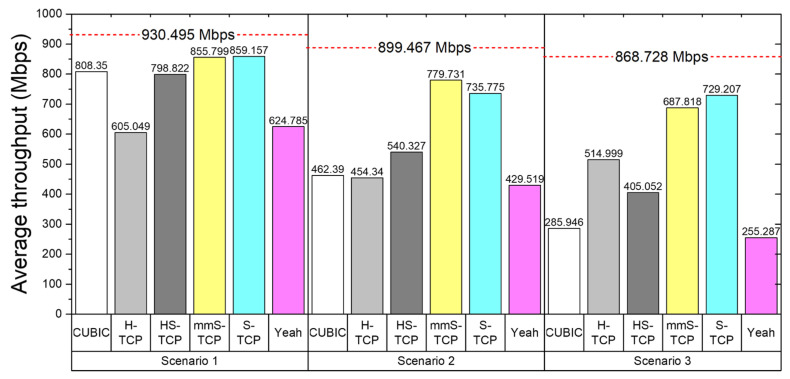
Comparisons of average throughput according to the scenarios and TCP CCAs.

Although S-TCP can provide sufficient throughput in mmWave links, it can generate many duplicated ACKs and cause fairness issues for other CCAs. In the mmS-TCP proposed in this study, the cwnd increase mechanism was changed, and a gentler cwnd increase than in S-TCP might have affected throughput. Therefore, we evaluated whether mmS-TCP could guarantee sufficient average throughput, and we compared average throughput of existing CCAs together. The average throughput of a single TCP flow was evaluated for three scenarios, and the optimal delivery rate for each scenario is displayed at the top in Figure 10.

In Scenario 1, where one instantaneous outage occurs, CUBIC TCP performed a total of two cwnd reductions in the initial stage of congestion avoidance and in the outage event. Thereafter, due to the slow increase in the congestion avoidance phase, the average throughput was about 808 Mbps. HS-TCP caused many duplicated ACKs in slowstart phase and decreased cwnd by a larger margin in the outage event due to the aggressive cwnd increase. This resulted in lower average throughput than CUBIC. H-TCP showed a relatively significant cwnd decrease and a slow cwnd increase in the congestion avoidance, and H-TCP took a long time to increase after RTO occurred in the outage event and cwnd decreased to 1. The Yeah algorithm stayed in congestion avoidance until the 15 s time mark, did not reach the maximum available capacity, and showed low average throughput due to the very slow cwnd increase mechanism. S-TCP and mmS-TCP occupied the available bandwidth more efficiently than the existing CCAs, and even when RTO occurred in an outage event, high average throughput was secured with a rapid increase in cwnd.

The average throughput of all CCAs decreased in Scenarios 2 and 3, where multiple large and small NLoS transmissions occurred. However, S-TCP and mmS-TCP had the smallest average throughput reduction. Although there was a difference in average throughput between S-TCP and mmS-TCP depending on the scenario, both congestion controls ensured average throughput of about 80% or more of the available capacity. None of the remaining CCAs provided sufficient average throughput due to repeated slow congestion avoidance after reducing cwnd with frequent NLoS events.

### 4.5. Inter-Protocol Fairness (vs. CUBIC)

In mmWave networks, an aggressive increase in cwnd can improve TCP throughput, but it can cause fairness issues for other flows. Therefore, an inter-protocol fairness experiment was performed to investigate whether S-TCP and mmS-TCP, which provide high throughput, compete fairly with the existing CCAs. The fairness with CUBIC, which was adopted as the default congestion control in all representative operating systems such as Linux, Windows, and MAC, was evaluated.

In a scenario in which three NLoS events occur, it was assumed that a single CUBIC flow competes one-to-one on the mmWave link provided by the same base station as CUBIC, S-TCP, and mmS-TCP. In addition, evaluation of the competition situation between S-TCP and mmS-TCP was also performed.

**Figure 11:**
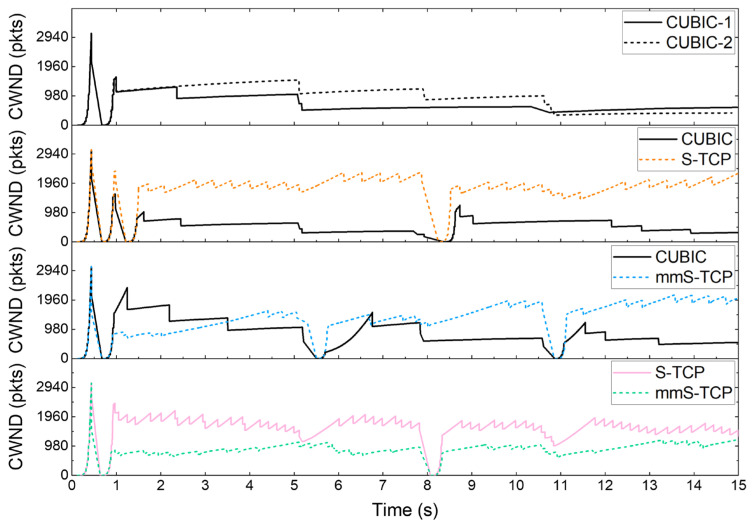
Cwnd variation in competition between two flows.

**Figure 12:**
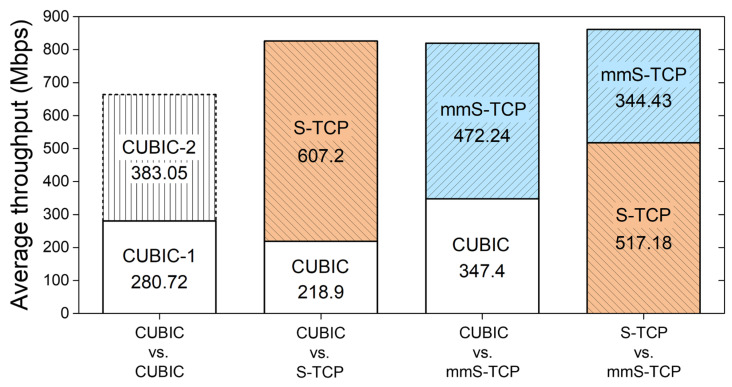
Average throughput of each flow when they compete.

Figure 11 shows the change of cwnd over time, and Figure 12 shows the average throughput of each flow as stacked bars. When two CUBIC flows compete, the flow that reduces cwnd first results in lower throughput. This is not because of the aggressiveness of a specific flow, but because cwnd increases very slowly in the congestion avoidance of the CUBIC algorithm. Two CUBIC flows that do not fully utilize the capacity of the mmWave link cannot reduce the difference in cwnd until a new packet drop occurs. The cwnd of the two flows become similar due to continuous packet drops occurring in the CUBIC_2 flow, but the cwnd after 11 s is of a size that cannot fully utilize the link.

When S-TCP competes with CUBIC flow, many packet losses occur at the beginning of the connection with two slowstart phases. S-TCP immediately rises based on the ssthresh value set in the second slowstart, while CUBIC does not rise high because it is set lower than S-TCP. The gap between the two flows, which occurs immediately after the slowstart, is temporarily narrowed at the time the RTO occurs (about 8 s), but the gap widens again due to S-TCP’s aggressiveness.

Unlike S-TCP, mmS-TCP is not dominant from the beginning of the connection because it sets the ssthresh similar to that of CUBIC after one slowstart. After 11 s of an RTO event, mmS-TCP dominates, but if an NLoS situation triggers an RTO, mmS-TCP halves the ssthresh, giving the CUBIC flow a chance to reverse. At the bottom of Figure 11, we can see the cwnd fluctuations when S-TCP and mmS-TCP compete. Due to the aggressiveness of S-TCP, mmS-TCP showed relatively low cwnd size than S-TCP. However, mmS-TCP can compete with S-TCP to provide better throughput compared to CUBIC.

Figure 12 shows the average throughput of each flow over 15 s of competition. In the competition between the two CUBIC flows, one flow has the upper hand and the total throughput is low, as it does not fully use the available capacity of the link. Total throughput is higher when S-TCP and mmS-TCP compete with CUBIC, but S-TCP shows approximately 2.8 times higher throughput than CUBIC flow. mmS-TCP also has higher throughput than the competing CUBIC flow, but the throughput of the CUBIC flow increases about 1.6-fold compared with the case of CUBIC vs. CUBIC. mmS-TCP had a throughput of 344.43 Mbps when competing with S-TCP and slightly overwhelmed by S-TCP but showed approximately 50% higher throughput than when CUBIC competed with S-TCP.

In a situation where the CUBIC, S-TCP, and mmS-TCP algorithms compete with each other, S-TCP occupies a lot of bandwidth and it was confirmed that S-TCP harms inter-protocol fairness (average throughput; S-TCP: 494 Mbps, mmS-TCP: 264 Mbps, CUBIC: 88 Mbps).

For Jain’s fairness index [40], CUBIC, S-TCP, and mmS-TCP were 0.9768, 0.819, and 0.9773, respectively, and mmS-TCP showed the highest fairness index. The redesigned mmS-TCP did not ideally share the link with CUBIC TCP, but mmS-TCP improves fairness with CUBIC than S-TCP without compromising total throughput. In addition, mmS-TCP competed with S-TCP and provided a relatively high fairness index of 0.9614.

### 4.6. Intra-Protocol Fairness

TCP congestion control must fairly occupy the link capacity, not only in competition between different algorithms, but also in competition between identical algorithms. Following the fairness evaluation for CUBIC TCP, Section 4.6 compares the fairness within the protocol.

The intra-protocol fairness experiments assumed a scenario in which the UE moves for 30 s and experiences a total of five NLoS situations at intervals of about 5 s. Here, five TCP flows were tested in a topology through which one UE was connected to five different servers and passed through one mmWave base station.

**Figure 13:**
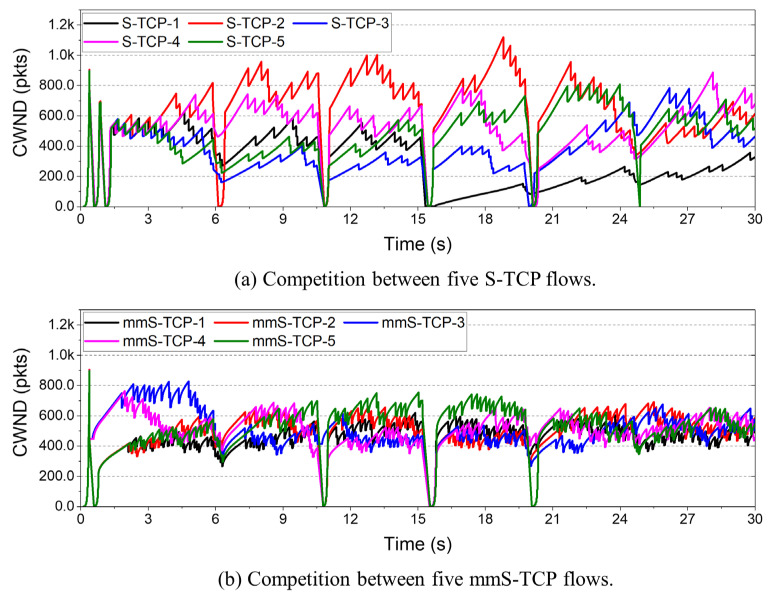
Cwnd changes in competition between five flows: (**a**) S-TCP; (**b**) mmS-TCP.

Figure 13a shows the change in cwnd in the competition between five S-TCP flows, and Figure 13b shows the result of mmS-TCP. S-TCP is very dense at the beginning of the connection, but soon shows a higher cwnd increase in certain flows. The flow that is initially dominant overwhelms the others for a long time. The specific S-TCP flow does not increase to the ssthresh after the NLoS situation, and congestion avoidance is performed at a low cwnd. Therefore, it can be seen that S-TCP is somewhat unstable in competition with the same protocol, and an imbalance in throughput occurs.

On the other hand, for the mmS-TCP of Figure 13b, some flows dominate at the beginning of the connection, but eventually the cwnd values of all flows closely converge. mmS-TCP has smaller cwnd fluctuations between flows than does S-TCP, and it competes with others within a relatively narrow range. These results are due to the redesign of the cwnd growth mechanism, which increases rapidly in the initial stage and slowly increases in the last stage. This makes convergence easier to reach during competition between the same protocols.

**Figure 14:**
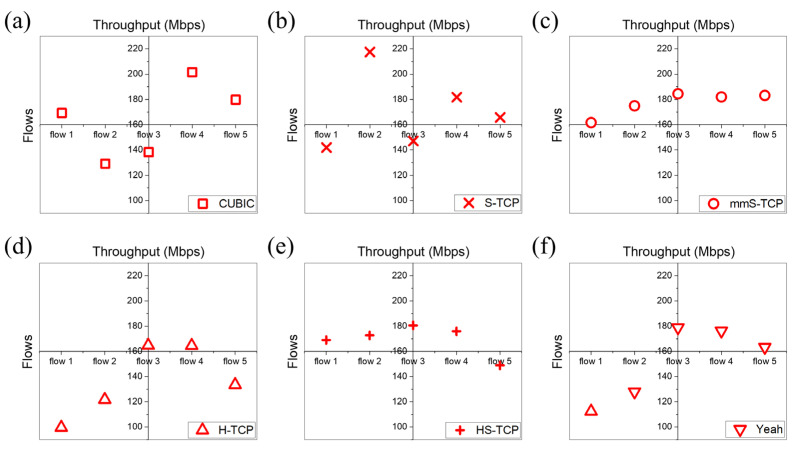
Throughput comparison of five flows according to the CCAs: (**a**) CUBIC; (**b**) S-TCP; (**c**) mmS-TCP; (**d**) H-TCP; (**e**) HS-TCP; (**f**) Yeah.

For an additional intra-protocol fairness comparison, Figure 14 shows the throughput of each flow for the six TCP CCAs evaluated in Section 4.4. mmS-TCP and HS-TCP show relatively similar throughput between flows, but the remaining CCAs show a large difference in throughput between the five flows. Table 3 shows the Jain’s fairness index values between the five flows according to CCAs, the throughput of each flow, and the average total throughput. mmS-TCP simultaneously improves average total throughput and intra-protocol fairness through the redesign of the cwnd increase/decrease mechanism.

## 5. Discussion

mmS-TCP is based on NewReno, a conventional ACK-based congestion control algorithm. Therefore, the RTT fairness problem, which is the biggest drawback of ACK-based CCAs, may occur (the performance of a flow with a short RTT is high). However, the time-based CUBIC TCP, which improved on the shortcomings of existing CCA, may also be affected by RTT and cause fairness issues [41]. Of course, the fact that it is based on the classic CCA is a clear disadvantage; to compensate for this, we are considering a CUBIC-based CCA design for mmWave networks in our future work.

X-TCP [25] and MillProxy [24], introduced in Section 2, have the disadvantage of being difficult to design and deploy. In particular, because both proposals are based on a cross-layer method, there are difficulties in their actual implementation. Meanwhile, the redesigned mmS-TCP in this study has the advantage that it is able to provide sufficient performance in mmWave networks through a simple modification of the existing algorithm, S-TCP, and that it is easy to design and deploy.

Before understanding the behavior of TCP on mmWave links, important RAN parameters must also be considered. To support a high-speed wireless network, the deployment of multiple micro base stations is required rather than the wide coverage of a large signal tower. The dense deployment of micro base stations and fast handovers between base stations present various challenges for transport layer protocols. In particular, the handover mechanism is a factor that can greatly affect end-to-end performance depending on user mobility. Additionally, because the mmWave band has already been used for satellite communication, it can be expected that the terrestrial and satellite communication frequency bands will gradually overlap [9]. Therefore, as the use of the mmWave band expands, consideration should begin as to whether to use existing transport layer protocols for integrated communications.

## 6. Conclusions

In this paper, we proposed mmS-TCP to improve TCP performance over mmWave links. Existing TCP does not provide sufficient throughput due to low link utilization in the highly variable mmWave channel. Depending on the queue management scheme applied to mmWave base stations, throughput may increase, but RTT will also increase, so the design goal of 5G cannot be achieved.

Therefore, in order to achieve the 5G design goal of high delivery rate and ultra-low latency at the same time, the existing TCP congestion control algorithm was redesigned to increase link utilization, and the AQM scheme was adopted to lower end-to-end delay. First, by modifying the congestion window increase/decrease mechanism of S-TCP, we improved the stability of TCP flow while providing high throughput. In addition, the CoDel queue management scheme was applied to mmWave base stations to prevent the bufferbloat problem, thereby lowering end-to-end delays.

We implemented several simulation scenarios based on NS-3 to evaluate the performance of the redesigned mmS-TCP in the mmWave networks. The simulation results showed that mmS-TCP reduced the total number of duplicated ACKs to half compared with S-TCP and reduced the number of duplicated ACKs to 1/4 in the slowstart phase. In addition, mmS-TCP guaranteed throughput greater than 80% of the available capacity of mmWave links.

In the evaluation of fairness between protocols, S-TCP, which showed a fairness index of 0.819, greatly overwhelmed CUBIC, whereas mmS-TCP competed relatively fairly with CUBIC with a fairness index of 0.9733. Even in the evaluation of intra-protocol fairness between five identical protocols, mmS-TCP simultaneously improved average total throughput and intra-protocol fairness. In future work, we plan to implement a CUBIC-based congestion control algorithm that can provide sufficient performance in mmWave networks to compensate for the shortcomings of the ACK-based algorithm.

## Figures and Tables

**Table 1:** Simulation parameters.

	Carrier Frequency	Bandwidth	TxPower	RLC Buffer Size	RLC ACK Mode	Hybrid ARQ	TCP RTO	Queue Management	Simulation Time
Set value	28 GHz	1 GHz	30 dBm	4 MB	Enabled	Enabled	200 ms	CoDel	15 s

Notes: TxPower: transmitted power; RLC: radio link control; ACK: acknowledgement; ARQ: automatic repeat request; TCP: transmission control protocol; RTO: retransmission time-out.

**Table 2:** Comparisons of the rate of duplicated ACKs to total packet.

	Scenario 1		Scenario 2		Scenario 3	
	Total Dup. ACKs/Total Packets	Dup. ACKs (≤3.0 s)/Total Packets	Total Dup. ACKs/Total Packets	Dup. ACKs (≤3.0 s)/Total Packets	Total Dup. ACKs/Total Packets	Dup. ACKs (≤3.0 s)/Total Packets
S-TCP	9.7%	6.4%	11.4%	7.4%	11.5%	7.5%
mmS-TCP	5.0%	1.8%	5.8%	2.3%	6.1%	2.6%
CUBIC	0.56%	0.35%	1.92%	0.84%	2.23%	1.35%
HS-TCP	4.65%	3.82%	7.66%	6.63%	10.26%	8.84%
H-TCP	2.97%	2.22%	3.65%	3.1%	3.32%	2.74%
Yeah	0.13%	0.13%	0.19%	0.19%	0.67%	0.32%

Notes: Dup. ACKs: duplicated acknowledgements; S-TCP: scalable TCP; mmS-TCP: scalable TCP for mmWave; HS-TCP: highspeed TCP; H-TCP: Hamilton TCP; TCP: transmission control protocol.

**Table 3:** Comparisons of Jain’s fairness index, the throughput of each flow, and the average total throughput.

	Flow 1 [Mbps]	Flow 2 [Mbps]	Flow 3 [Mbps]	Flow 4 [Mbps]	Flow 5 [Mbps]	Average Total Throughput [Mbps]	Jain’s Fairness Index [Ratio]
S-TCP	141.8775	217.5223	147.088	181.7001	165.7131	853.901	0.975083
mmS-TCP	161.6046	174.928	184.4512	181.9639	183.1542	886.102	0.997718
CUBIC	169.239	129.0921	138.1982	201.5095	179.7331	817.772	0.974036
HS-TCP	168.977	172.6625	180.5496	175.9145	148.8744	846.978	0.995843
H-TCP	99.5897	121.7243	164.707	164.5096	133.5064	684.037	0.967242
Yeah	112.3956	127.9662	178.8458	176.2211	163.2704	758.699	0.96984

Notes: S-TCP: scalable TCP; mmS-TCP: scalable TCP for mmWave; HS-TCP: highspeed TCP; H-TCP: Hamilton TCP; TCP: transmission control protocol.
